# Cleaved cytokeratin-18 is a mechanistically informative biomarker in idiopathic pulmonary fibrosis

**DOI:** 10.1186/1465-9921-13-105

**Published:** 2012-11-20

**Authors:** Seung-Ick Cha, Christopher J Ryerson, Joyce S Lee, Jasleen Kukreja, Sophia S Barry, Kirk D Jones, Brett M Elicker, Dong Soon Kim, Feroz R Papa, Harold R Collard, Paul J Wolters

**Affiliations:** 1 Department of Medicine, University of California, San Francisco School of Medicine, Box 0111, San Francisco, 94143-0111, CA, USA; 2 Department of Internal Medicine, Kyungpook National University School of Medicine, Daegu, South Korea; 3 Department of Surgery, University of California, San Francisco, CA, USA; 4 Department of Pathology, University of California, San Francisco, CA, USA; 5 Department of Radiology, University of California, San Francisco, CA, USA; 6 Department of Medicine, Asan Medical Center, University of Ulsan College of Medicine, Seoul, South Korea

**Keywords:** Idiopathic interstitial pneumonia, idiopathic pulmonary fibrosis, lung fibrosis, ER stress, apoptosis

## Abstract

**Background:**

Stress of the endoplasmic reticulum (ER) leading to activation of the unfolded protein response (UPR) and alveolar epithelial cell (AEC) apoptosis may play a role in the pathogenesis of idiopathic pulmonary fibrosis (IPF). Our objectives were to determine whether circulating caspase-cleaved cytokeratin-18 (cCK-18) is a marker of AEC apoptosis in IPF, define the relationship of cCK-18 with activation of the UPR, and assess its utility as a diagnostic biomarker.

**Methods:**

IPF and normal lung tissues were stained with the antibody (M30) that specifically binds cCK-18. The relationship between markers of the UPR and cCK-18 was determined in AECs exposed in vitro to thapsigargin to induce ER stress. cCK-18 was measured in serum from subjects with IPF, hypersensitivity pneumonitis (HP), nonspecific interstitial pneumonia (NSIP), and control subjects.

**Results:**

cCK-18 immunoreactivity was present in AECs of IPF lung, but not in control subjects. Markers of the UPR (phosphorylated IRE-1α and spliced XBP-1) were more highly expressed in IPF type II AECs than in normal type II AECs. Phosphorylated IRE-1α and cCK-18 increased following thapsigargin-induced ER stress. Serum cCK-18 level distinguished IPF from diseased and control subjects. Serum cCK-18 was not associated with disease severity or outcome.

**Conclusions:**

cCK-18 may be a marker of AEC apoptosis and UPR activation in patients with IPF. Circulating levels of cCK-18 are increased in patients with IPF and cCK-18 may be a useful diagnostic biomarker.

## Background

Idiopathic pulmonary fibrosis (IPF) is a progressive interstitial lung disease (ILD) with no established pharmacotherapy and a median survival of 3 years [1,2]. Due to incompletely understood pathophysiology and the lack of mechanistic biomarkers, drug development and testing in IPF typically relies on relatively insensitive clinical measures such as change in pulmonary function or survival time. Identification of mechanistically informative biomarkers of specific pathologic processes may allow for faster, cheaper, and safer development and testing of candidate drugs.

Several studies have implicated type II alveolar epithelial cell (AEC) injury and death in the pathogenesis of IPF [3-7]. In some instances, epithelial cell death is programmable, as AEC apoptosis has been demonstrated in patients with IPF [3-5]. Proposed causes of AEC apoptosis in IPF include oxidative stress and DNA damage, [8] TGF-β and Fas ligand, [9] and stress of the endoplasmic reticulum (ER). ER stress occurs when there is an imbalance between cellular demand for protein synthesis by the ER and the ER’s capacity to synthesize, process and package the requisite proteins [5,6]. When under stress, the ER attempts to maintain homeostasis by activating the unfolded protein response (UPR) pathway [10]. If the UPR fails to restore homeostasis, it sacrifices the cell by activating apoptotic pathways [11]. Markers of the UPR and apoptosis are increased in lung tissue from patients with IPF and localize to the alveolar epithelium [5,6]. These observations suggest ER stress and activation of the UPR may be one trigger of AEC apoptosis in IPF patients.

Cytokeratin-18 is a cytoskeletal protein that is primarily found in pseudostratified and simple epithelia, including the alveolar epithelium [12]. During apoptosis of epithelial cells, cytokeratin-18 is cleaved twice by caspases, generating an 18-kilodalton fragment termed caspase-cleaved cytokeratin-18 (cCK-18). This caspase-specific processing exposes a neo-epitope at the c-terminal end of cCK-18 that is recognized by a specific monoclonal antibody (M30) [13]. The overall objective of this study was to use the M30 antibody to define whether there is a relationship between activation of the UPR and formation of cCK-18 in lung AECs, and to determine whether circulating cCK-18 could serve as a mechanistic biomarker of the UPR and AEC apoptosis in IPF.

## Methods

### Type II alveolar epithelial cell isolation and induction of ER Stress

Type II AECs were isolated from explanted IPF lungs and unused donor lungs (procured from the Northern California Transplant Donor Network) as previously described [14]. Purity of human alveolar type II cells, assessed by pro-surfactant protein C (SPC) staining on cytospin, was > 95% for IPF and normal lungs. The diagnosis of IPF was confirmed in all cases by multidisciplinary review according to established criteria [15,16]. A549 cells were obtained from the American Type Culture Collection (Manassas, VA, USA). Cells were exposed to thapsigargin (EMD Chemicals, Inc., Gibbstown, NJ, USA) for varying lengths of time to induce ER stress.

### Lung tissue preparation and immunohistochemistry for cCK-18

For immunohistochemistry, IPF lung tissue was obtained from the Tissue Core Laboratory of the Lung Tissue Research Consortium (Denver, CO). Healthy normal lung tissue was procured from lungs not used by the Northern California Transplant Donor Network. Briefly, endogenous peroxidase was inhibited in 5-μm tissue sections by incubating the sections in 3% hydrogen peroxide for 30 minutes. After washing with PBS, the sections were incubated for one hour in PBS containing 5% normal goat serum and 1% bovine serum albumin (BSA), then incubated with a 1:500 dilution of monoclonal mouse anti-cCK18 antibody (M30 antibody, M30 CytoDEATH, PEVIVA, Bromma, Sweden) overnight at 4°C. The sections were washed in 0.1% PBS-Tween 20, incubated for 40 minutes with horseradish peroxidase (HRP)-conjugated goat anti-mouse IgG (Santa Cruz Biotechnology, Santa Cruz, CA, USA). After washing, the bound peroxidase activity was detected using a diaminobenzidine (DAB) substrate kit (Vector Laboratories, Burlingame, CA, USA). The sections were then washed in deionized water and counterstained with Meyer’s hematoxylin (Sigma-Aldrich). For each tissue, an adjacent section was stained only with secondary antibody to address nonspecific binding of the secondary antibody.

### FACS Sorting

Epithelial cells were suspended in DME H-21 containing 10% fetal bovine serum and incubated for 30 minutes on ice with antibodies to Annexin V (BD Biosciences, San Jose CA) per the manufacturer’s protocol. Samples were then analyzed using a LRS II flow cytometer (Becton Dickinson) and sorted into Annexin V + or - populations using a Moflo High Performance Cell Sorter (Dako Cymation).

### Immunoblots

Cell lysates were subjected to SDS-PAGE under reducing conditions and transferred to nitrocellulose membrane (Life Sciences Products, Boston, MA). The membrane was washed with 50 mM Tris–HCl containing 0.5 M NaCl, 0.01% Tween-20, (TBS; pH 7.5) and incubated overnight in 5% milk containing primary antibody. The membrane was then washed with TBS, incubated in TBS for 30 min containing a 1:2,000 dilution of HRP-conjugated secondary antibody (New England Biolabs, Beverly, MA) and washed again. Immunoreactivity was detected using the phototope-HRP-detection kit (New England Biolabs). Primary antibodies included: mouse anti-human cCK-18 (1:1000, M30), rabbit-anti human IRE-1α (1:1000 Novus Biologicals, Littleton, CO, USA), rabbit-anti human phospho-specific IRE-1α (1:2000, Novus Biologicals), and mouse anti-human β-actin (1:1000, Sigma-Aldrich).

### XBP-1 mRNA splicing

Total RNA from type II AECs and A549 cells was extracted using a standard Trizol (Invitrogen, Carlsbad, CA) protocol. cDNA was synthesized from total RNA using One-Step RT-PCR SuperScript III reverse transcriptase (Invitrogen) per the manufacturer’s protocol. XBP-1 splicing was quantified by PCR using methods described previously [17]. Sense primer mXBP1.3S (5’- AAACAGAGTAGCAGCGCAGACTGC-3’) and antisense primer mXBP1.2AS (5’- GGATCTCTAAAACTAGAGGCTTGGTG-3’) were used. PCR fragments were digested by PstI. The PstI cleavage site is located in the 26 nt intron of unspliced XBP-1, which allows differentiation between the unspliced XBP-1 amplicon (cut PCR product) and the spliced XBP-1 amplicon. PCR products were resolved on 2% agarose gels, stained with EtBr, and quantified by densitometry. XBP1-specific primers were synthesized by Integrated DNA Technologies Inc (Coralville, IA, USA). The PCR fragments were visualized with BioDoc-It Imaging System (UVP, Inc., Upland, CA, USA).

### Blood and BAL ELISA for cCK-18

Blood samples, clinical information, and survival data from patients with IPF, chronic hypersensitivity pneumonitis (HP), and nonspecific interstitial pneumonitis (NSIP) enrolled in an on-going longitudinal research cohort were collected at the time of initial presentation to UCSF. All ILD diagnoses were established thorough multidisciplinary review of clinical data, radiology, and pathology according to established criteria[15,16,18,19]. Patients with chronic HP required either a diagnostic surgical lung biopsy, or a compatible HRCT combined with a chronic and high-intensity exposure (e.g. living with a pet bird, living or working on a farm). Patients with NSIP were defined according to previously published criteria [20]. Bronchoalveolar lavage (BAL) samples were obtained for research purposes in a subset of IPF subjects. A second cohort consisted of blood samples from stable IPF and acute exacerbation of IPF patients seen at Asan Medical Center, University of Ulsan (Seoul, South Korea) [21]. Stable IPF patients were defined as outpatients with IPF who were not experiencing a rapid decline in respiratory function. Acute exacerbation of IPF was diagnosed using consensus criteria [22]. Written informed consent was obtained from all patients according to institutional review board-approved protocols. In all samples, M30 reactivity was measured using a commercially available ELISA (M30-Apoptosense ELISA, PEVIVA, Bromma, Sweden) according to the manufacturer’s protocol.

### Statistical analysis

Serum cCK-18 levels were compared between IPF and control groups using the Wilcoxon rank-sum test. The association of cCK-18 level with the diagnosis of IPF was tested for confounding by age, gender, smoking history, dyspnea severity, the use of long-term supplemental oxygen therapy, baseline FVC, and baseline DLCO using regression analysis. The ability of serum cCK-18 level to predict disease progression in the UCSF cohort was tested using a Wilcoxon rank-sum test. Disease progression was indicated as previously defined by our group: 10% decline in FVC, 15% decline in DLCO, lung transplantation, or death due to any cause within 6 months of the blood draw [23]. Multivariate Cox proportional hazards analysis was used to evaluate the relationship of cCK-18 with transplant-free survival. All analyses were repeated using a dichotomous cCK-18 variable. All data analysis was performed using STATA 11.0 (StataCorp, Texas, USA).

## Results

### The UPR is activated in type II alveolar epithelial cells isolated from IPF lung

**Figure 1:**
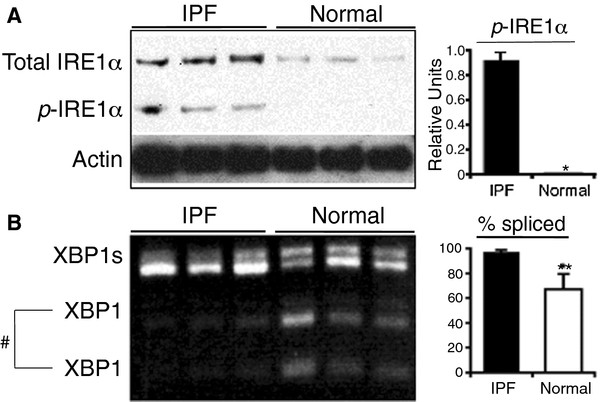
**The UPR is activated in type II AECs isolated from IPF lungs.** Phosphorylated IRE-1α (panel **A**) and spliced XBP-1 (panel **B**) were present in increased amounts in AECs from 3 different IPF lungs, but not in the 3 control subjects (* P < 0.05, **P = 0.0495 by Wilcoxon rank sum test). # indicates amplicon of unspliced XBP1 digested with Pst1.

It has been reported that the UPR is activated in the epithelial cells of IPF patients. To confirm these observations, the relative levels of phospho-IRE-1α, and XBP-1 mRNA splicing were measured in AECs isolated from normal and IPF lungs. Type II AECs of IPF lungs expressed more total IRE-1α compared to normal lungs and the phosphorylated form was detected in type II AECs of IPF, but not in normal lungs (Figure 1A). There was also more spliced XBP-1 in type II AECs of IPF lungs compared to normal lungs (p = 0.0495; Figure 1B). These findings demonstrate that the UPR pathway is activated in type II AECs isolated from IPF patients.

### ER stress triggers cCK-18 formation in cultured alveolar epithelial cells

**Figure 2:**
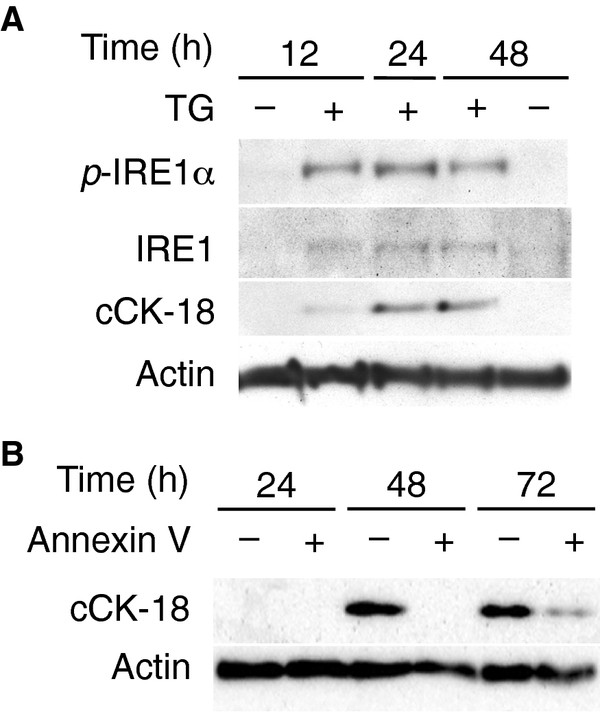
**cCK-18 is formed following activation of the UPR. A**. Immunoblots for phospho-IRE1 (p-IRE1), total IRE1 (IRE1), and cleaved cytokeratin 18 (cCK-18) showing time-dependent increase of cCK-18 in A549 cells exposed to 0.5 μM of thapsigargin (TG, +) for various lengths of time. Immunoblots showing an increase in total and phospho-IRE1 confirm thapsigargin activates the UPR in A549 cells compared to vehicle controls (−). **B**. Immunoblots for cCK-18 showing time-dependent appearance of cCK-18 in annexin V^–^ or V^+^ A549 cells exposed to 0.5 μM of thapsigargin for various lengths of time.

Because ER stress and subsequent activation of the UPR can lead to apoptosis, [11] we next examined whether induction of ER stress leads to the formation of cCK-18. To do this, A549 cells were exposed to thapsigargin, an inducer of ER stress, for varying lengths of time. Phosphorylated IRE-1α expression increased following thapsigargin exposure (Figure 2A), confirming that thapsigargin activated the UPR in AECs. cCK-18 increased in thapsigargin-exposed A549 cells (Figure 2A), demonstrating that activation of the UPR can lead to formation of cCK-18 in AECs.

### cCK-18 is formed prior to the appearance of the apoptosis marker Annexin V

Next we measured the appearance of thapsigargin-mediated cCK-18 formation in relationship to annexin V, an early marker of apoptosis. Thapsigargin-exposed A549 cells were harvested and separated into two populations (annexin V + or –) by flow sorting for the cell surface expression of annexin V. Figure 2B shows that cCK-18 first appears in annexin V^–^ cells 48 hrs after thapsigargin exposure, and remains present in annexin V^–^ cells 72 hrs after thapsigargin, when it begins to appear in annexin V^+^ cells.

### cCK-18 is present in the alveolar epithelium of IPF lung

**Figure 3:**
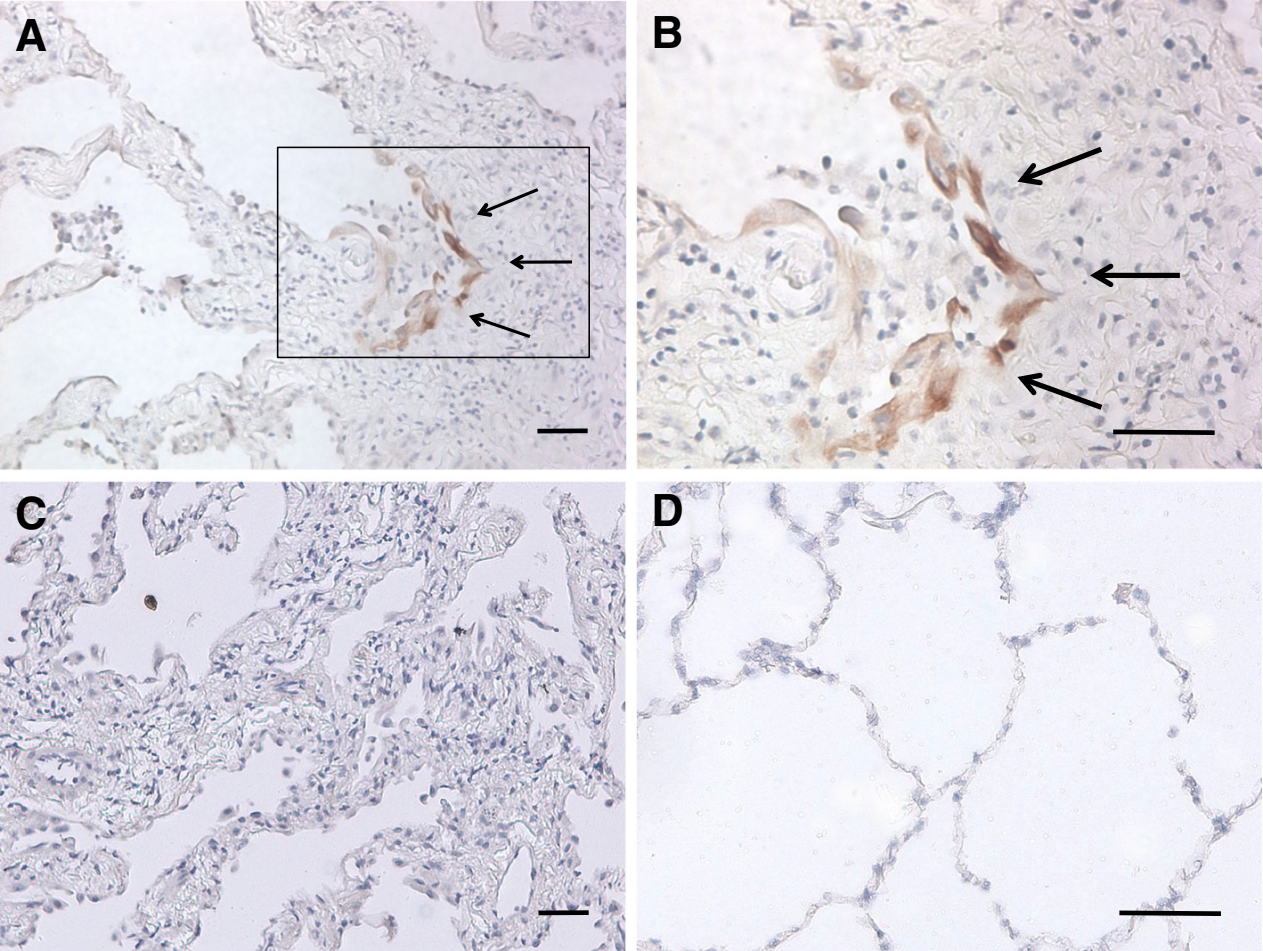
**Immunohistochemistry of IPF and normal lung with M30 antibody. A**. IPF lung immunostained with the M30 antibody (20x). **B**. Higher power view of IPF lung immunostained with the M30 antibody (40x). **C**. IPF lung immunostained with a non-immune antibody (20x). **D**. Normal lung immunostained with the M30 antibody showing an absence of staining for cCK-18 (40x). Bar = 50 μm. Images are representative of staining of tissue from 8 patients with IPF and 8 control subjects.

Activation of the UPR and apoptosis have been reported in AECs of IPF lungs[5,6]. To examine whether cCK-18 formation is similarly present in IPF AECs, sections of lung obtained from IPF patients were immunostained for cCK-18. AECs of IPF lung were immunoreactive for cCK-18 (Figures 3A, B). In contrast, lung tissue from control subjects was not immunoreactive for cCK-18 (Figure 3D).

### cCK-18 is elevated in the serum of IPF patients

**Figure 4:**
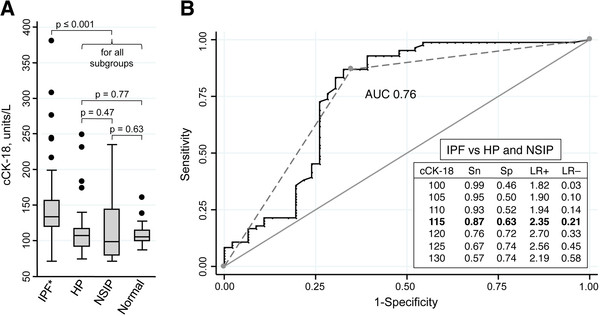
**Serum cCK-18 levels in patients with lung fibrosis. A**. Serum cCK-18 level is higher in IPF (n=84) than HP (n=24), NSIP (n=22), or control subjects (n=39); p ≤ 0.001 for comparison of IPF with all subgroups). cCK-18 level is similar in HP, NSIP, and control subjects. **B**. cCK-18 distinguishes IPF from other fibrotic ILDs (area under the receiver operating characteristic curve [AUC] = 0.76). Sensitivity (Sn), specificity (Sp), and likelihood ratios (LR) are shown for the diagnosis of IPF (versus HP and NSIP) using several cCK-18 thresholds. A cut-off of 115 units per liter (dashed line) provided the highest AUC (0.76). * Four outliers are excluded from the IPF group for illustrative purposes (cCK-18 values not shown: 433, 513, 979, 1147 units/L).

cCK-18 has been reported to be a circulating biomarker of epithelial cell apoptosis [12]. To examine whether circulating cCK-18 levels are preferentially increased in patients with IPF, cCK-18 levels were measured in the serum of 169 subjects, including 84 subjects with IPF, 24 with HP, 22 with NSIP, and 39 control subjects. Demographic and clinical characteristics of these subjects are summarized in Table 1. Serum cCK-18 levels were significantly elevated in the serum of IPF patients compared to control subjects (area under the receiver operating characteristic curve (AUC) 0.88, p<0.00005) (Figure 4A). Furthermore, serum levels of cCK-18 were significantly different in IPF patients compared to those in patients with HP or NSIP (AUC 0.76, p<0.00005) (Figure 4B). There were no differences in cCK-18 level comparing HP, NSIP, and control subjects. cCK-18 was an independent predictor of a diagnosis of IPF versus HP and NSIP when controlling for baseline variables (p=0.001). The addition of cCK18 to baseline variables significantly improved the AUC from 0.83 to 0.88 (p<0.00005). cCK-18 was not detectable in BAL fluid of IPF patients.

**Table 1:** Subject characteristics of UCSF cohort

**Variable**	**IPF**	**HP**	**NSIP**
	**(n = 84)**	**(n = 24)**	**(n = 22)**
Age, years	70.3 (7.9)	57.0 (10.3)*	63.6 (12.5)*
Male sex, %	76.2	33.3*	59.1
Diagnosis by surgical lung biopsy, %	50.0	66.7	72.7
**Smoking history**			
Ever smoked, %	78.6	41.7*	31.8*
Pack-years	27.7 (28.7)	11.8 (18.8)*	6.4 (13.8)*
**Measures of disease severity**			
Dyspnea score	9.8 (6.0)	11.2 (4.6)	9.6 (4.4)
Long-term oxygen therapy, %	20.2	23.8	27.3
Pulmonary function			
FVC, % predicted	69.4 (18.2)	67.3 (22.0)	64.1 (19.1)
DLCO, % predicted	46.7 (18.4)	55.1 (21.2)	43.7 (14.4)

Data shown are mean (standard deviation), unless otherwise indicated.

* p < 0.05 for comparison with the IPF cohort.

Abbreviations: DLCO, diffusing capacity of carbon monoxide; FVC, forced vital capacity; HP, hypersensitivity pneumonitis; IPF, idiopathic pulmonary fibrosis; NSIP, nonspecific interstitial pneumonia; UCSF, University of California San Francisco.

### Serum cCK-18 level is not associated with clinical variables or outcomes

**Figure 5:**
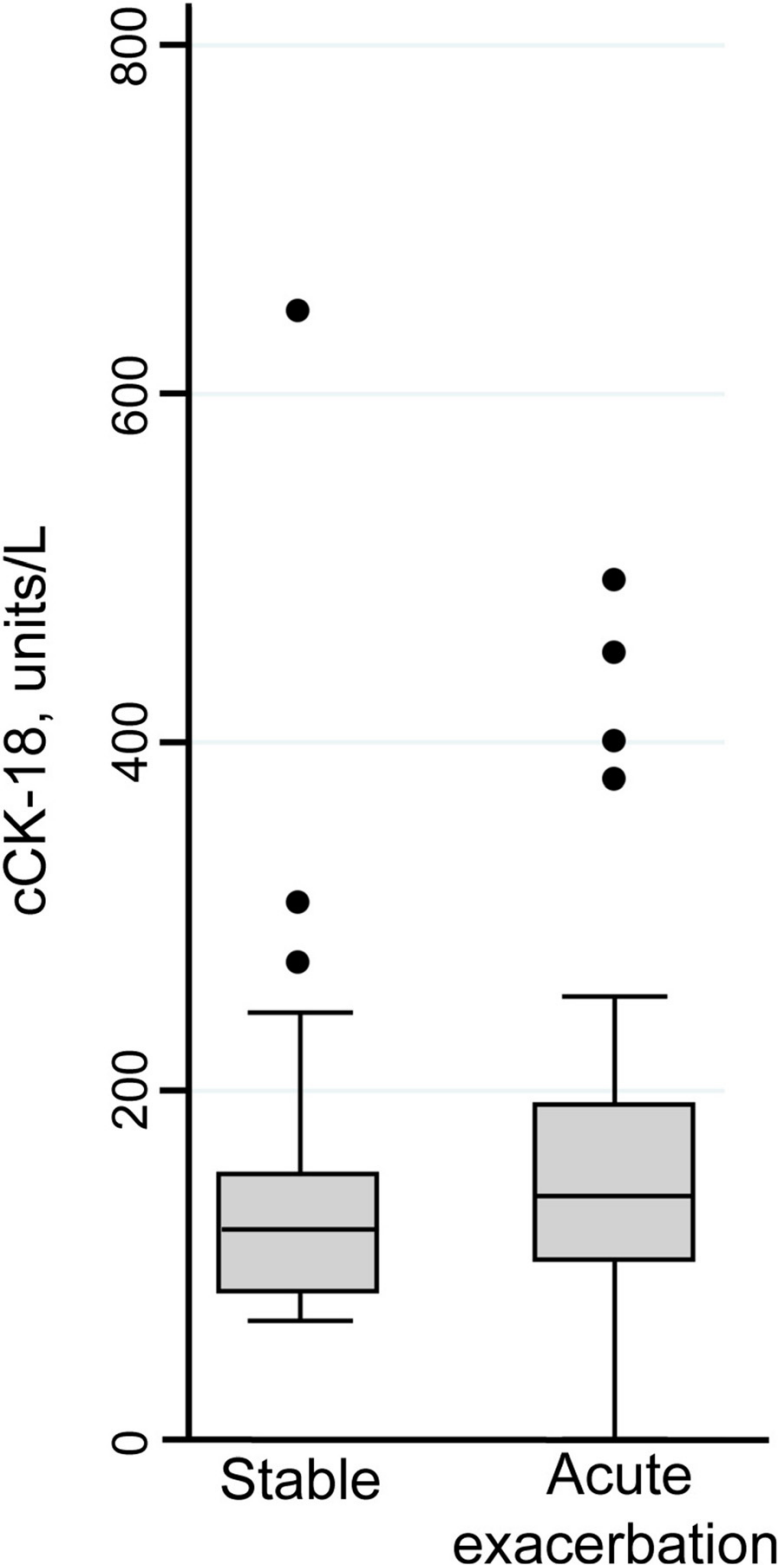
**Serum cCK-18 is not increased during acute exacerbation of IPF.** Serum cCK-18 levels were measured in the serum of 34 patients with stable IPF and 36 different patients suffering from an acute exacerbation of IPF.

Serum cCK-18 level was not associated with any baseline clinical variables. Further, serum cCK-18 level did not predict progression measured by a change in FVC, DLCO or survival. There was no change in results with secondary analyses that analyzed cCK-18 as a dichotomous variable or that considered alternative outcome variables (i.e. considering individual pulmonary function test variables as dichotomous or continuous variables). In a separate cohort of patients, cCK-18 level was not increased during acute exacerbation of IPF compared to stable disease (p = 0.15) (Figure 5).

## Discussion

We have shown that markers of ER stress and the UPR are increased in type II AECs isolated from IPF lung, confirming that these pathways are activated in IPF patients. We have also shown that cCK-18 is present in IPF AECs, is generated by activation of the UPR in vitro, and is uniquely elevated in the serum of patients with IPF compared to normal and diseased ILD control subjects. This suggests that cCK-18 may be a biomarker of the UPR and AEC apoptosis and that it could be used to monitor therapies modulating the UPR or AEC apoptosis in IPF patients.

The UPR is a mechanism by which the ER attempts to maintain homeostasis when exposed to proteins that are unfolded or folded incorrectly [10]. Initially, the UPR attempts to restore homeostasis within the ER. Failure to restore homeostasis via the UPR leads to apoptosis [11]. Previous studies have shown that the UPR is activated in the alveolar epithelium of human IPF lung [5,6]. We build upon these data by directly confirming that the UPR is activated in IPF AECs and that circulating levels of cCK-18, a cleavage product of cytokeratin 18 formed during the UPR, is elevated in IPF patients.

A mechanistically informative biomarker should reflect the activity of important biological pathways. Previous biomarker studies in IPF have focused more on measuring levels of proteins that predict prognosis rather than measuring proteins whose levels are predictive of specific pathologic processes; examples include surfactant protein-A and-D (SP-A and SP-D), [24-26] MMP7, [27,28] and Krebs von den Lungen 6 antigen (KL-6), [29,30]. CC-chemokine ligand 18 (CCL18) is an additional biomarker that is increased in IPF serum and bronchoalveolar lavage fluid and may predict progression[31-33]. CCL18 is a marker of alternative macrophage activation and stimulates collagen production in normal lung tissue in vitro; [34,35] however a clear mechanistic role of CCL18 in IPF has not been proven. Unlike previous biomarkers, this study investigates the use of cCK-18 as a marker of the UPR and apoptosis in IPF patients, two pathologic processes that are activated in IPF patients [4-6]. cCK-18 is generated by caspases activated during apoptosis of epithelial cells, [12] suggesting it is a marker for AEC apoptosis in patients with IPF. In addition, new data in this manuscript (Figure 2) show that cCK-18 is formed following activation of the UPR in lung epithelial cells. These findings suggest that cCK-18 may be a marker for apoptosis or the UPR in IPF patients.

Circulating cCK-18 does not distinguish between activation of the UPR and apoptosis in epithelial cells. Nevertheless, data in Figure 2B demonstrate that epithelial cells generate cCK-18 following activation of the UPR yet before expression of the apoptosis marker annexin V. This suggests cCK-18 may be generated in cells containing levels of active caspase that are insufficient to activate apoptosis, but sufficient to cleave cytokeratin 18. Because the number of alveolar epithelial cells that are immunoreactive for cCK-18 (Figure 3) or active caspase 3 [8] are far greater than the rare TUNEL positive alveolar epithelial cell found in IPF lungs (data not shown), this is the most likely scenario in IPF lung. Proving this will require future identification of more specific biomarkers of the UPR that can be correlated to cCK-18.

We show that cCK-18 could also be a useful diagnostic biomarker that distinguishes IPF from chronic HP and NSIP, independent of age, gender, smoking, disease severity, and other baseline variables. A recent small study found that cCK-18 may also be elevated in the serum of patients with organizing pneumonia [36]. The clinical importance of this finding is uncertain, given that IPF and organizing pneumonia have unique clinical and radiological features [19]. We used chronic HP and idiopathic fibrotic NSIP as disease controls because the clinical, radiologic, and pathologic features of chronic HP and fibrotic NSIP often have substantial overlap with IPF [15,16,18,19]. Distinguishing IPF from HP and NSIP often requires a surgical lung biopsy, a procedure that carries substantial risk [15,16]. If confirmed in other cohorts, a serum biomarker, such as cCK-18, that distinguishes IPF from other fibrotic ILDs may reduce the need for lung biopsy.

Serum cCK-18 was not associated with severity or progression of IPF. This conflicts with a previous study that showed cCK-18 correlated with physiologic measures in patients with a variety of ILDs [36]. Several possibilities could explain the lack of association seen our study. First, cCK-18 was measured at a single time point. This single measurement may not be representative of activation of the UPR or apoptosis over the course of an individual’s disease. Second, circulating levels of cCK-18 likely reflect the sum of a complex interplay of physiological processes (i.e. production, clearance, metabolism), each of which may impact the cCK-18 level differently in individual patients. In addition, physiologic progression was measured over a time interval of 6-months after cCK-18 measurement, and cCK-18 level might reflect disease activity and progression on a more limited time scale (e.g. days or weeks). Third, although data were adjusted for several clinical and physiologic variables, other confounders may have been present, including occult conditions, unrelated to IPF, that could cause apoptosis of AECs. cCK-18 was also not elevated in the serum of patients during acute exacerbation of IPF. This may suggest that AEC apoptosis is not a prominent feature of acute exacerbation, however this finding requires further study as there was a trend for higher serum levels of cCK-18 in patients during acute exacerbation of IPF. Finally, we also were unable to detect cCK-18 in BAL fluid in patients with IPF. The reason for this is unknown, but may relate to dilution that occurs during the BAL procedure or to different metabolism of cCK-18 in the alveoli compared to the serum.

## Conclusions

In summary, data reported in this manuscript show that the UPR is activated in AECs of IPF patients and that cCK-18 may be a marker of this process. This finding suggests that cCK-18 could be used in the early-phase development of drugs targeting the UPR in IPF patients. In addition, serum cCK-18 levels may be clinically informative as a diagnostic marker of IPF. Further research measuring serial cCK-18 levels in a longitudinal cohort of IPF patients is required to confirm these results and determine the impact of temporal changes in serum cCK-18 on its performance as a measure of disease activity and course.

## Abbreviations

AEC: Alveolar epithelial cell; AUC: Area under the receiver operating characteristic curve; BAL: Bronchoalveolar lavage; BSA: Bovine serum albumin; cCK-18: Caspase-cleaved cytokeratin-18; CCL18: CC-chemokine ligand 18; DAB: Diaminobenzidine; DLCO: Diffusing capacity for carbon monoxide; DNA: Deoxyribonucleic acid; ELISA: Enzyme-linked immunosorbent assay; ER: Endoplasmic reticulum; FACS: Fluorescence-activated cell sorting; FVC: Forced vital capacity; HP: Hypersensitivity pneumonitis; HRP: Horseradish peroxidase; ILD: Interstitial lung disease; IPF: Idiopathic pulmonary fibrosis; KL-6: Krebs von den Lungen 6 antigen; M30: Monoclonal antibody to cCK-18; MMP7: Matrix metalloproteinase 7; NSIP: Nonspecific interstitial pneumonia; PBS: Phosphate buffered saline; RNA: Ribonucleic acid; RT-PCR: Reverse transcriptase polymerase chain reaction; SDS-PAGE: Sodium dodecyl sulfate polyacrylamide gel electrophoresis; SP-A: Surfactant protein A; SP-D: Surfactant protein D; TBS: Tris-buffered saline; TGF-β: Transforming growth factor-beta; TUNEL: Terminal deoxynucleotidyl transferase dUTP nick end labeling; UCSF: University of California San Francisco; UPR: Unfolded protein response; XBP-1: X-box binding protein 1.

## Competing interests

The authors have no competing interests related to this manuscript.

## Author contributions

SIC performed the experiments and produced the first draft of the manuscript. CJR performed data analysis, contributed to some of the experiments, and produced the first draft of the manuscript. JSL contributed bronchoalveolar lavage samples. JK contributed tissue samples. SSB performed experiments. KDJ reviewed pathology for all ILD patients. BME reviewed radiology for all ILD patients. DSK provided samples for patients with acute exacerbation of IPF. FRP participated in the study design and data analysis. HRC participated in the study design and data analysis. PJW conceived of the study, oversaw the experiments, and produced the first draft of the manuscript. All authors read and approved the final manuscript.
